# Cholecysto-Intestinal Fistula for the Relief of Occlusion of the Common Bile Duct

**Published:** 1888-02

**Authors:** 


CHOLECYSTO-INTESTINAL FISTULA FOR THE RELIEF OF OCCLUSION OF THE COMMON BILE DUCT.—A notice of a successful case of cholecystenterostomy, by Kappeler, in the *Medical Record*, of November 12th, gives an erroneous impression of his proceeding, in stating that "the operation consists of emptying the gall-bladder and stitching an opening in its wall, directly to a corresponding slit in the duodenum."

Upon examining the original report, it is found that he distinctly states his inability to reach the duodenum, and that he availed himself of a coil of the small intestine, into which an incision was made, to which he sutured the incision primarily made through the walls of the gall-bladder after its contents were evacuated by a trocar.

This modification of Winiwarters' operation by completing the union at once, instead of adopting his primary and secondary steps, should be accepted as a satisfactory solution of the problem, restoring the bile to the alimentary canal in cases of occlusion of the common bile duct; and no one can doubt henceforth the practicability of this surgical procedure. Notwithstanding Kappeler's impression of the danger of uniting the gall-bladder and duodenum, the question whether the communication should be made with the small intestine below the duodenum, or with the walls of the duodenum, as suggested by Dr. J. McF. Gaston, Atlanta, Ga., remains *subjudice*.

In view of the advantages likely to be gained by conveying the bile into the alimentary canal, near the point of its normal entrance through the ductus choledochus, and the proximity of this portion of the duodenum to the gall-bladder, it seems proper to bring to the attention of surgeons the following points presented in the various publications of Dr. Gaston touching this measure of cholecysto-intestinal fistula:

In his original article on obstruction of the gall-duct, which appeared in this journal October, 1884, Gaston states that "it is proper, under some circumstances, to evacuate the distended gall-bladder externally, with a view to establish subsequently a free communication from it to the intestinal canal, which is requisite for a successful result of the operation. Having made an incision into the peritoneal cavity, over the most prominent part of the distension, a digital or ocular examination may enable the operator to determine upon the propriety of opening the sac," (p. 379). "A finger may be introduced, and bring the surface of the alimentary canal below the insertion of the duct in contact with a portion of the wall of the sac, so that a needle with an elastic ligature may be passed through the tissues of both, having their adjoining surfaces united closely in the loop which constricts the inclosed walls until they are cut through, so as to leave a communication between the two, such as has been observed in the ulcerated opening of the gall-badder into the upper portion of the small intestine," (p. 380).

"On the 20th of August, 1884, a puppy of three or four months was operated on, under the influence of ether, by making an incision of two and a half inches, and passing a silk ligature through a very small portion of the walls of the gall-bladder and duodenum, by which they were secured in close contact," (p. 381).

"On the 28th the subject of this experiment was found to be apparently free from all trouble, and exploration revealed union of the gall-bladder and duodenum, and no doubt existed of the exit by opening between the gall-bladder and the duodenum, as the small size of the gall-bladder indicated that it was kept drained," (p. 383).

In an article, entitled "Duodeno-Cholecystotomy," contributed by Dr. Gaston to the second volume of the "Reference Hand-book of the Medical Sciences," he says: "My conviction became fixed, in the course of the year 1881, that surgical means should be adopted with a view to an artificial opening between the gall-bladder and the duodenum, or the adjacent portion of the small intestine, and my article substantially as published, with exception of the experiments on dogs, was sent to *Gaillard's Journal.*

The proximity of the duodenum to the under-surface of the gall-bladder presents favorable conditions when there is distention of the gall-bladder with more or less thickening of its walls, in cases of obstruction of the common duct in the human subject. There are three features in this operative measure which are essential to a successful issue: The firm union of the exterior surfaces of the walls that are brought in contact; a free opening through both structures by which their cavities are in communication; the removal of the suture after cutting its way by a spontaneous passage into the intestinal canal. So far as our experiments upon the dog are capable of illustrating what might be expected in the human being, we have every reason to expect a favorable result," (p. 540). Six dogs were used in this first series of experiments.

"The fatality attending the ligature of the common bile-duct before another outlet for the accumulation of bile was effected induced me to undertake three experiments for effecting an immediate communication between the gall-blader and the duodenum. With proper precaution in removing a larger section from the duodenal wall so as to make allowance for its contractility, and a secure union of its margin with that of the opening in the sac, this operation ought to succeed," (p. 543).

Another series of experiments on fifteen dogs, published in the ATLANTA MEDICAL AND SURGICAL JOURNAL for the months of September and November, 1885, illustrated this cholecysto-duodenal outlet for the bile in obstruction of the common duct, and "the result of attachment of the gall-bladder to the duodenum by a single loop of white silk thread, are their intimate and firm union, by adhesive inflammation between their serous surfaces, and the formation of a fistulous opening through this septum, which affords a communication between their cavities."

The investigations of Golzi, reported June 12, 1886, in the *Central-blatt für Chirurgie*, as to the establishment of a union between the gall-bladder and duodenum, in obstruction of the common bile duct, are referred to by Dr. Gaston in his paper laid before the British Medical Association at Brighton. His experiments proved the practicability of attaching the gall-bladder and duodenum by suturing an orifice in each, so as to secure the mucous and serous membranes in apposition, and the operation led to no trouble either in the gall-bladder or duodenum, thus confirming Gaston's claim.

In the same paper, re-published in this journal, on the practicability of establishing an artificial fistulous opening in the human subject between the gall-bladder and duodenum, Dr. Gaston remarks as follows:

"In case of an incision into the sac for the removal of its solid contents, this may be closed by Lembert's suture, separate from the loop of attachment, or may be secured to a corresponding incision in the duodenum, dispensing thus with the loop for effecting a communication."

When speaking of Harley's suggestion as compared with his own, it is stated that "some other process may, however, be found preferable to either, and it rests with surgeons to adopt that which proves best. My experiments on dogs were undertaken to verify a predetermined method of making an opening from the gall-bladder into the duodenum, and it only remains to test its applicability to man for this operation to be adopted."

Such are the facts for consideration by those who may encounter cases of occlusion of the common bile duct requiring an operation.—*Gaillard's Medical Journal.*

